# Window Coverage and Liquid Biopsy in the First-Line Therapy of Severe Sudden Sensorineural Hearing Loss

**DOI:** 10.3390/brainsci14111154

**Published:** 2024-11-19

**Authors:** Alexander Kilgue, Rayoung Kim, Lars-Uwe Scholtz, Conrad Riemann, Christoph J. Pfeiffer, Matthias Schürmann, Ingo Todt

**Affiliations:** Department of Otolaryngology, Head and Neck Surgery, Campus Klinikum Bielefeld Mitte, Medical School OWL, Bielefeld University, Teutoburger Str. 50, 33604 Bielefeld, Germany; alexander.kilgue@klinikumbielefeld.de (A.K.); rayoung.kim@klinikumbielefeld.de (R.K.); lars-uwe.scholtz@klinikumbielefeld.de (L.-U.S.); conrad.riemann@klinikumbielefeld.de (C.R.); christoph.pfeiffer@klinikumbielefeld.de (C.J.P.);

**Keywords:** cochlin-tomoprotein (CTP), sudden sensorineural hearing loss (SSNHL), perilymphatic fistula (PLF), tympanoscopy

## Abstract

Introduction: Based on clinical practice guidelines, the application of corticosteroids as a first-line therapy is common. Although sudden sensorineural hearing loss (SSHL) etiology is primarily idiopathic, hearing loss can result from a perilymphatic fistula (PLF). Recent findings show evidence of a specific rate of PLF based on a cochlin-tomoprotein (CTP) detection test. Based on this rate of PLF treatment, the concepts of SSNHL need to be re-evaluated. The present study aimed to evaluate CTP in SSNHL patients and compare pre-surgical and post-surgical pure tone hearing thresholds after round and oval window sealing as first-line treatment. Material and Methods: A total of 30 patients with unilateral SSNHL with a pure tone average (PTA) (4 Freq. of 60 dB or more were enrolled in a prospective study. All patients underwent tympanoscopy for middle ear exploration as a first-line treatment. After intraoperative observation of a possible PLF, all patients obtained middle ear lavage to gain CTP samples for following ELISA-based CTP detection tests. All patients received round window and oval window sealing with fascia. PTA hearing thresholds were analyzed post-surgically 3 weeks after treatment based on 4-frequency bone conduction (BC). Results: The average preoperative pure tone BC threshold was 97.7 dB compared with the 69 dB postoperative BC threshold. Mean BC improved by 20.3 dB after middle ear exploration and window sealing. A total of 56% (17 of 30) of patients recovered at least 10 dB. The middle ear cochlin-tomoprotein detection rate was 70% positive. Conclusions: The combination of early tympanoscopy and inner ear-specific cochlin-tomoprotein as a detection tool for suspected PLF showed evidence of PLF as a key causative in SSNHL.

## 1. Introduction

Sudden sensorineural hearing loss (SSNHL) is characterized by an acute, mostly unilaterally occurring inner ear dysfunction. It is defined as a hearing function decline of at least 30 dB in three sequential frequencies [1]. Frequently but not universally accompanied symptoms can be vertigo, tinnitus, and ear fullness with individual patterns of occurrence [2].

The incidence of SSNHL is estimated to range from 5 to 400/100,000, depending on the sensitivity of observation. Pathophysiological mechanisms include vascular disorders, viral and bacterial infections, rheological dysfunction, neoplasms, and autoimmune diseases, among other etiologies, such as pressure changes in the endolymphatic and perilymphatic systems of the inner ear [3].

Mostly regarded as idiopathic, SSNHL etiology and pathogenesis remain unclear to date. The treatment of SSNHL remains controversial and highly discussed. According to clinical practice guidelines, treatment options for SSNHL vary and a “universal” standard procedure has not yet been established [4]. The application of systemic and/or intratympanic corticosteroids in various dosages as a first-line therapy of SSNHL is a common standard occasionally in combination with histamines and vasodilators, although it is still a matter of debate as to what degree these therapy concepts outrun spontaneous recovery [5].

Although the etiology of SSNHL is mostly considered idiopathic, membrane ruptures of the round and/or oval window with consecutive perilymphatic fistula (PLF) are described as a cause of sudden hearing loss, tinnitus, and vertigo. Membrane ruptures might occur after head trauma due to pressure alterations, iatrogenic procedures, or anatomical malformations. Spontaneous membrane rupture is also described [6,7].

The consideration of PLF as a possible cause of SSNHL has been known for decades. Surgical sealing of the round and/or oval window membrane via exploratory tympanotomy as a diagnostic and therapeutic tool has been applied and established since the 1970s but still remains highly discussed [8,9,10]. To date, there is no consensus on performing exploratory tympanotomy and window membrane sealing, although PLF has reached importance in etiology in the case of SSNHL [11].

So far, no consensus exists on diagnostic criteria for PLF. Intraoperatively diagnosing PLF while performing exploratory tympanotomy can be difficult. Leakage of perilymph from the round or oval window might not be visible when tympanotomy is performed. False fibrous membranes of the round window can make intraoperative visual interpretation hence PLF difficult to diagnose. Visible fluid might be challenging when distinguishing PLF from intraoperative local fluid injection, such as local anesthesia [6]. Intraoperative tests such as pressure transmission tests and fistula tests lack evidence of reliability. Reliable noninvasive tests have not yet been established. Objective diagnostic tests with beta-trace protein or fluorescein staining have been described but lack specificity and are technically ambitious to perform [12,13,14].

Ikezono et al. identified the inner ear-specific cochlin-tomoprotein (CTP), which is specific and sensitive to perilymph. This protein can be used to objectively assess potential PLF using a monoclonal antibody test [15].

Recent findings show evidence of a 22%/47% PLF rate based on the CTP detection test in cases of SSNHL [16,17]. Based on this high rate of PLF, SSNHL treatment concepts need to be re-evaluated.

This study aimed to detect CTP in SSNHL patients and compare pre-surgical and post-surgical pure tone hearing thresholds after round and oval window sealing as a first-line treatment.

## 2. Materials and Methods

### 2.1. Sample Collection and Evaluation of CTP Concentration

We collected CTP samples by performing middle ear cavity lavage with 0.3 mL of saline and absorption of the fluid, which is defined as middle ear lavage (MEL). The MEL was centrifuged (6000 rpm for 15 s; Eppendorf Systems, Hamburg, Germany). The supernatant liquid was collected and stored at −20 °C. The samples were evaluated for CTP by monoclonal antibody testing and ELISA CTP (TECAN/JBL: 301170068) was performed. Cutoff values for monoclonal antibody ELISA CTP were the following: <30 ng/mL indicated no evidence of PLF, 30–60 ng/mL indicated intermediate evidence of PLF, and >60 ng/mL indicated evidence of PLF.

### 2.2. Subjects

Study group:

We prospectively analyzed patients treated for severe to profound unilateral sudden sensorineural hearing loss with tympanotomy and consecutive round and oval window membrane sealing as a first-line treatment in our department between November 2022 and October 2023. We defined severe to profound SSNHL as a hearing function decline of 60 dB or more in three consecutive frequencies. Head computed tomography (CT) and magnet resonance tomography imaging (MRI) assessments were performed to exclude other possible reasons for acute hearing loss. The indication for tympanotomy as a first-line treatment was SSNHL with a mean PTA/bone conduction of at least 60 dB. Surgical treatment was followed by 3 days of i.v. steroids (250 mg prednisolone).

Exclusion criteria were patterns of other diseases explanatory for SSNHL and an age of under 18 years. Individual data are collected in Table 1.

### 2.3. Surgery

Explorative tympanotomy was performed under general anesthesia. After endaural incision and tympanomeatal flap preparation, middle ear exploration and intraoperative assessment of possible PLF was carried out. Partial bone removal was performed to obtain a complete overview of the oval window. The round and oval windows were identified. The ossicle chain and stapes footplate were assessed. Via applying pressure, movement of the ossicle chain was provoked, and fluid leaks of the round and oval window membranes were evaluated. All patients immediately obtained middle ear lavage to gain CTP samples for following ELISA-based CTP detection tests. A total of 0.3 mL of saline was applied into the tympanic cavity, and absorption occurred via syringe after fluid rinsing three times. Regardless of the intraoperative assessment, both oval and round windows were obturated. All patients received first-line treatment with round window and oval window sealing with fascia obtained from tissue from the endaural incision. Additionally, fibrin glue was applied to optimize window sealing. The applied surgical technique was similar in every operation.

### 2.4. Pure Tone Audiometry

PTA hearing thresholds were analyzed pre-surgically (baseline) and post-surgically 21 days after treatment (Auritec AT 1000, Hamburg, Germany). We assessed the mean hearing thresholds based on the three most affected contiguous frequencies between 0.5 and 4 kHz (Max 3 Freq ^0 = 110 dB^, maximum hearing loss set 0 = 110 dB) and the mean 4-frequency bone conduction at 0.5, 1, 1.5, and 4 kHz (4 Freq ^0 = 130 dB^, maximum hearing loss set 0 = 130 dB). Recovery was defined as a mean change in PTA BC of at least 10 dB. Failure to respond was defined as an improvement in BC less than 10 dB—a classification of the hearing threshold according to WHO hearing impairment [18].

### 2.5. Vestibular Symptoms

Classification occurred based on the absence or presence of vertigo at the onset of hearing impairment.

### 2.6. Intraoperative Findings

Visual assessment of the ossicular chain, round, and oval window membranes was conducted at microscopic magnification. The examination regarded integrity and fluid leaks. The ossicle chain and stapes footplate were assessed. The ossicle chain movement was provoked by applying pressure, and fluid leaks of the round and oval window membranes were checked. A positive PLF was classified as findings when a window membrane rupture was detected and/or fluid in the round and oval membrane areas and/or dislocation of the stapes was noticed.

## 3. Results

### 3.1. Patient Characteristics

Between November 2022 and October 2023, 30 patients with severe to profound unilateral sudden sensorineural hearing loss underwent tympanotomy and consecutive sealing of their round and oval window membranes as first-line treatment. All patients underwent complete ENT examinations, including CT head scans, MRIs, and pure tone audiometry.

Preoperative age range varied from 18 to 89 years (mean 55.9 years). A total of 17 patients were male (57%), while 13 patients were female (43%). Explorative tympanotomy was performed within a range of 1 to 16 days after the onset of symptoms (median 5.0, mean 5.8, standard deviation 4.2 days).

Mostly, evaluation of SSNHL etiology based on the patient’s information upon admission indicated an idiopathic origin in 27 out of 30 cases (90%), as patients could not name any reason for their hearing impairment in their medical history or in-house conducted diagnostics led to other plausible origins. In only three cases (10%), a traumatic event (pressure trauma, head injury) was associated with SSNHL. Vestibular symptoms occurred in 53% (*n* = 16). In two out of three cases (67%) with traumatic history, vestibular symptoms were present. Idiopathic cases had vestibular symptoms in 14 of 27 cases (52%) (For individual data, see Table 1).

### 3.2. Surgery

Surgery was performed after a mean of 5.8 days upon the occurrence of symptoms with a range between 1 and 16 days (median 5.0, mean 5.8, standard deviation 4.2 days). A total of 30 patients underwent explorative tympanotomy with consecutive sealing of the round and oval window membranes as a first-line treatment of severe to profound SSNHL. Surgery was performed under general anesthesia.

### 3.3. Hearing Outcomes After Surgery

Hearing recovery was defined as a mean improvement in PTA bone conduction (BC) of at least 10 dB from the baseline PTA (preoperative) to PTA on day 21. Failure to respond was an improvement in BC less than 10 dB.

The mean PTA hearing threshold at admission to the hospital was preoperatively 97.7 dB (Max 3 Freq ^0 = 110 dB^) and 104.6 dB (4 Freq ^0 = 130 dB^) on the affected ear.

After explorative tympanotomy with consecutive sealing of the round and oval window membranes, 57% (17 of 30) of patients improved by at least 10 dB in postoperative PTA Max 3 Freq ^0 = 110 dB^.

A total of 53% (16 of 30) of patients improved by at least 15 dB in postoperative PTA Max 3 Freq ^0 = 110 dB^. However, 43% (13 of 30) failed to respond with an improvement in BC less than 10 dB in postoperative PTA Max 3 Freq ^0 = 110 dB^.

PTA Max 3 Freq ^0 = 110 dB^ improvement of 10–20 dB (mean 17.4 dB) was observed in five cases. Improvement of 20–30 dB (mean 26. dB) occurred in five cases. Two cases improved between 30 and 40 dB (mean 34.5 dB). Furthermore, five cases improved more than 40 dB (mean 69.7 dB) after window coverage.

A total of 63% (19 of 30) of patients improved by at least 10 dB in postoperative PTA 4 Freq ^0 = 130 dB^, and 60% (18 of 30) improved by at least 15 dB in postoperative PTA 4 Freq 0 = 130 dB. However, 37% (11 of 30) of patients failed to respond with an improvement in BC less than 10 dB in postoperative PTA 4 Freq 0 = 130 dB.

A PTA 4 Freq ^0 = 130 dB^ improvement of 10–20 dB (mean 21.6 dB) was observed in four cases. An improvement of 20–30 dB did not occur. Four cases improved between 30 and 40 dB (mean 33.4 dB). Furthermore, 11 cases improved more than 40 dB (mean 62.9 dB) after window coverage.

The mean hearing improvement in postoperative PTA 4 Freq ^0 = 130 dB^ was 27.8 dB, while the mean change in postoperative PTA Max 3 Freq ^0 = 110 dB^ was 20.3 dB.

**Figure 1:**
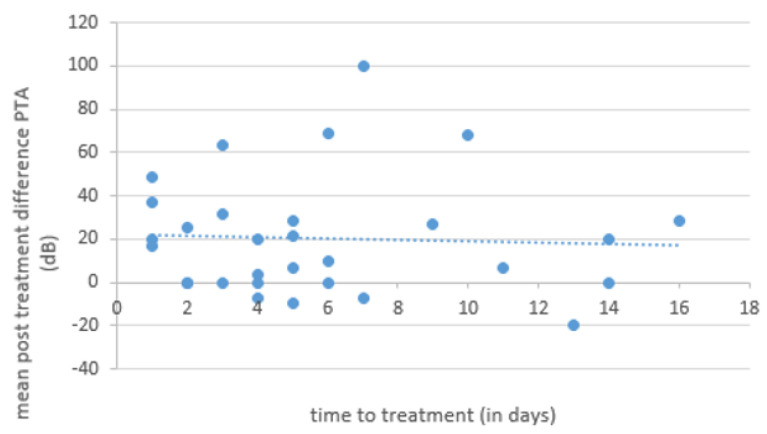
Relationship between time to treatment (in days) and mean postoperative difference in pure tone audiometry (PTA). Statistical analysis revealed no statistically significant association (Pearson’s correlation coefficient r = −0.05).

Statistical analysis revealed no statistically significant association between mean postoperative improvement in BC and time to treatment upon occurrence of symptoms (Figure 1).

### 3.4. Intraoperative Observation

The intraoperative observation was based on the surgeon’s subjective impression while inspecting the tympanic cavity. In 17 of 30 cases (57%), no abnormal findings were seen. Signs of a lesion of the round and/or oval window (fluid) occurred in 13 cases (43%). Mostly, we did not see an obvious lesion but clear fluid in the area of the window membrane. It is possible that the fluid was not perilymph but rinsing fluid or local anesthesia. Stapes movement was performed to evaluate fluid movement.

Three out of 13 cases (23%) with intraoperative signs of a PLF had a medical history of a traumatic event. In one case, the destruction of the footplate was detected. Ten of 13 cases (77%) with intraoperative signs of perilymph leak did not have a traumatic medical history and were classified as SSNHL of idiopathic origin.

### 3.5. CTP Results

A total of 70% (21 of 30) of SSNHL patients were CTP-positive according to CTP detection test cutoff criteria. Intermediate CTP was present in eight cases (27%) while one case was CTP-negative (3%). We compared the intraoperative subjective PLF evaluation with objective CTP test results. Five cases were false-positive, and 16 cases were false-negative. These findings can be concluded as having a sensitivity of 33.3% and a specificity of 16.7% for intraoperative subjective PLF evaluation.

Based on the enrolled patient’s information upon admission, 27 out of 30 cases (90%) indicated an idiopathic SSNHL origin. Out of the idiopathic origin group, 66.7% (*n* = 18) tested CTP-positive, 29.6% (*n* = 8) tested CTP-intermediate, and one case (3.7%) was classified as CTP-negative.

Three patients (10%) had a trauma etiology. All of the three patients (100%) with trauma etiologies tested CTP-positive.

In CTP-positive cases, we observed an improvement in BC > 10 dB (Max 3 Freq ^0 = 110 dB^) in 13 out of 21 cases (62%) and BC > 15 dB (Max 3 Freq ^0 = 110 dB^) in 12 cases (57%). The mean improvement in BC (Max 3 Freq ^0 = 110 dB^) in CTP-positive cases was 23.8 dB.

CTP-intermediate cases had a mean improvement of 13.6 dB BC (Max 3 Freq ^0 = 110 dB^). We observed an improvement in BC > 10 dB (Max 3 Freq ^0 = 110 dB^) in four out of eight cases (50%). These four cases (100%) had a BC improvement of >15 dB.

**Figure 2:**
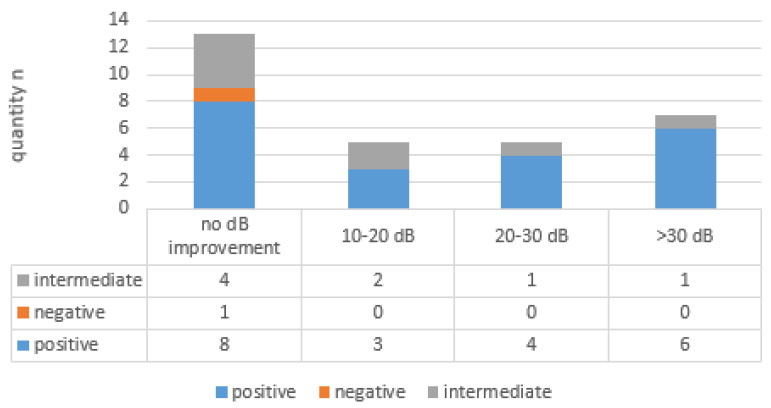
Comparison of bone conduction (BC) improvement in the three most affected contiguous frequencies between 0.5 and 4 kHz (Max 3 Freq ^0 = 110 dB^, maximum hearing loss set 0 = 110 dB) and the CTP category.

Max 3 Freq ^0 = 110 dB^ improvement in BC (see Figure 2) between 10 and 20 dB was CTP-positive in three out of five cases (60%) and CTP-intermediate in two out of five cases (40%). Improvement in BC between 20 and 30 dB was CTP-positive in four out of five cases (80%) and in one out of five cases (20%), CTP-intermediate. Improvement in BC > 30 dB was CTP-positive in six out of seven cases (86%) and CTP-intermediate in one out of seven cases (14%).

No BC improvement occurred in 13 out of 30 cases (43%), with eight CTP-positive cases, four CTP-intermediate cases, and one CTP-negative case.

The postoperative PTA 4 Freq ^0 = 130 dB^ showed an improvement in BC > 10 dB in 19 out of 30 cases (63%) in CTP-positive cases. We observed an improvement in BC > 15 dB (PTA 4 Freq 0 = 130 dB) in 18 out of 30 cases (60%). The mean improvement in BC (PTA 4 Freq 0 = 130 dB) in CTP-positive cases was 30.7 dB.

CTP-intermediate cases had a mean improvement of 24.0 dB BC (PTA 4 Freq ^0 = 130 dB^). An improvement in BC > 10 dB was observed in five out of eight cases (62.5%). These five cases (100%) had a BC improvement of >15 dB.

**Figure 3:**
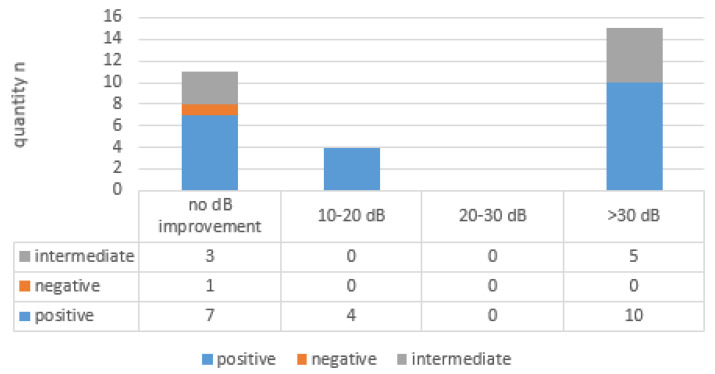
Comparison of bone conduction (BC) improvement in 4-frequency bone conduction at 0.5, 1, 1.5, and 4 kHz (4 Freq ^0 = 130 dB^, maximum hearing loss set 0 = 130 dB) and the CTP category.

PTA 4 Freq ^0 = 130 dB^ improvement in BC (see Figure 3) between 10 and 20 dB was CTP-positive in four out of four cases (100%). No improvement in BC between 20 and 30 dB occurred. Improvement in BC > 30 dB was CTP-positive in 10 out of 15 cases (67%) and CTP-intermediate in five out of 15 cases (33%).

No BC improvement occurred in 11 out of 30 cases (37%), with seven CTP-positive cases, three CTP-intermediate cases, and one CTP-negative case.

**Figure 4:**
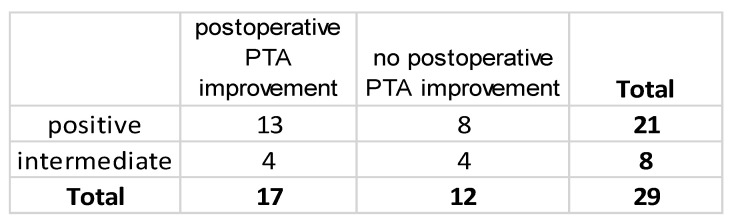
2 × 2 contingency table. Relationship between CTP categories (positive/intermediate) and postoperative PTA improvement. The association between CTP categories and postoperative PTA improvement was not statistically significant (Fisher’s exact test, *p* = 0.6828).

Statistical analysis revealed no statistically significant association between CTP categories (positive/intermediate) and postoperative PTA improvement (Figure 4).

**Figure 5:**
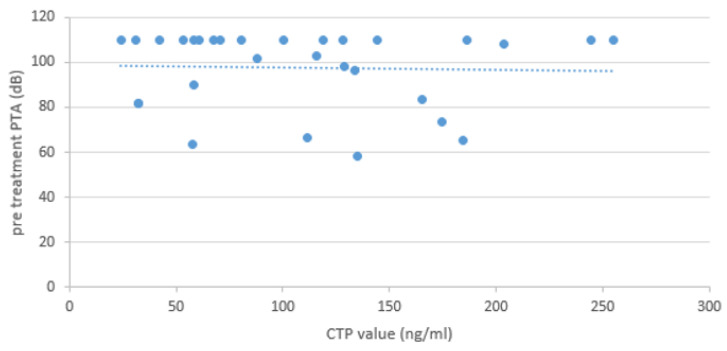
Correlation test of the individual CTP value (dots) and pre-treatment PTA. Statistical analysis showed no correlation between the CTP value and pre-treatment PTA (Pearson correlation).

We tested for correlation of the CTP value and pre-treatment PTA. Statistical analysis showed no correlation between the CTP value and pre-treatment PTA (Figure 5). CTP values ranged from 24.0 to 255.3 ng/mL. Cutoff values for monoclonal antibody ELISA CTP were the following: <30 ng/mL indicated no evidence of PLF, 30–60 ng/mL indicated intermediate evidence of PLF, and >60 ng/mL indicated evidence of PLF.

### 3.6. Vertigo

Vertigo occurred in addition to hearing loss in 16 out of 30 cases (53%). One out of 16 cases was CTP-negative (6%), while three out of 16 cases (19%) were associated with an intermediate CTP value. Twelve out of 16 cases (75%) were CTP-positive. In only two out of 12 positive CTP cases (17%), a trauma occurred according to the patient’s medical history. Out of 16 cases with vertigo, in nine cases, the PTA improved more than 10 dB postoperatively.

Vestibular testing was performed in CTP-positive cases. The results indicated that in all CTP-positive cases, there was vestibular receptor function impairment on the unilateral hearing loss-affected side.

## 4. Discussion

Membrane ruptures of the round and/or oval window with a consecutive perilymphatic fistula (PLF) are described as a cause of sudden hearing loss. Surgical sealing of the round and/or oval window membranes as a diagnostic and therapeutic tool remains highly discussed to date.

Intraoperative diagnosis of PLF while performing exploratory tympanotomy may lack objective evidence as visual confirmation might result from the surgeon’s subjective impression. Previously introduced PLF detection tests often lack evidence and are technically ambitious to perform [9]. Recent studies demonstrated high rates of PLF findings in SSNHL cases using explorative tympanotomy and consecutive objective PLF assessment via inner ear-specific cochlin-tomoprotein (CTP) testing [16,17].

We detected a high rate of CTP-positive PLF in 70% of the enrolled SSNHL patients. We compared intraoperative subjective PLF evaluation with objective CTP test results. Five cases were false-positive and 16 were false-negative, underlying CTP testing as the gold standard.

The etiology of the SSNHL patient group study cohort was 90% idiopathic. Only three cases (10%) recording traumatic events were associated with SSNHL. Our intraoperative observation, based on the surgeon’s subjective impression while inspecting the tympanic cavity, indicated normal findings in 57% of the cases.

Mostly, we did not see an obvious lesion, but clear fluid in the area of the window membrane was a possible indicator of a PLF. Despite visual “confirmation” of fluid as a possible indicator of a lesion of the round and/or oval window in 43% of cases, it is possible that the intraoperatively seen fluid was not perilymph but rinsing fluid or local anesthesia and therefore misinterpreted as PLF. As the intraoperative evaluation window during explorative tympanotomy highly depends on the surgeon’s subjective interpretation, there is a high degree of uncertainty.

Microruptures of the window membranes might not be visibly detectable as a PLF. Also, a fluctuating PLF due to a valve mechanism might occur. On the other hand, visible fluid from intraoperative local fluid injection, such as local anesthesia, might be misinterpreted as PLF [8].

We performed two-window sealing of both the oval and round windows. This is in line with the current literature in cases of severe to profound SSNHL [15]. After explorative tympanotomy with consecutive sealing of the round and oval window membranes, 57% (PTA Max 3 Freq ^0 = 110 dB^) and 63% (PTA 4 Freq ^0 = 130 dB^) of our study cohort improved by at least 10 dB.

We observed 70% CTP positivity according to the CTP detection test cutoff criteria in our SSNHL study cohort. In CTP-positive cases, we observed a postoperative improvement in BC > 10 dB in 62% (Max 3 Freq ^0 = 110 dB^), respectively, and 63% (PTA 4 Freq ^0 = 130 dB^) of cases. Mean improvement in BC in CTP-positive cases was 23.8 dB (Max 3 Freq ^0 = 110 dB^), respectively, and 30.7 dB (PTA 4 Freq ^0 = 130 dB^). CTP-intermediate cases had a mean improvement of 13.6 dB BC (Max 3 Freq ^0 = 110 dB^) and 24.0 dB BC (PTA 4 Freq ^0 = 130 dB^).

These findings indicate high evidence of perilymphatic fistula in severe SSNHL and a specific recovery rate after explorative tympanotomy and two-window sealing. The differences in BC improvement after window sealing between the CTP-positive, CTP-intermediate, and CTP-negative cases was logical as PLF repair through membrane sealing may reduce the negative impact on cochlear function and thereby improve postoperative bone conduction.

The only case of CTP negativity did not show any BC improvement at all. Neither the CTP value nor intraoperative findings indicated a PLF. Spontaneous PLF window sealing might explain the CTP negativity and normal intraoperative findings. Considered as an idiopathic etiology, it is important to take into account that this one case of CTP negativity already showed significant acute preoperative hearing loss. Due to the small sample size of one CTP-negative patient in our study group, the effect of window sealing in this patient group needs to be evaluated in a larger study cohort.

We observed vertigo in 57% of CTP-positive cases, which was additional to hearing impairment. These observations are in line with the literature, which correlates the occurrence of vertigo with the existence of a perilymphatic fistula [19].

No correlation was found between time to surgery and postoperative hearing recovery (Figure 1). Spontaneous PLF remission of the round and/or oval window in some PLF cases might be a possible explanation. It is important to note that the described spontaneous recovery rates in the literature include patients with mild hearing loss, low-degree hearing loss, and Ménière’s disease-linked low-frequency hearing loss with a higher potential of recovery [20]. These study cohorts differ substantially from our patient characteristics with severe to profound sudden sensorineural hearing loss and a generally significant weaker prognosis regarding hearing recovery according to the literature [21,22].

Further, there is a limited risk of increasing PLF-related hearing loss due to sucking at the RW and OW. Therefore, the procedure should be performed underwater. Additionally, a recurrent PLF fistula should be kept in mind related to a resorption of the covering tissue (e.g., fat, fascia).

PTA hearing thresholds were analyzed post-surgically 3 weeks after treatment. As a limitation of this study, a post-surgical 3-month evaluation is missing. PTA hearing recovery rates after 3-month evaluation are even expected to have been higher considering the positive effects of partial hearing recovery in this study after 3 weeks in >50% of patients. Further, a control group without intervention is missing to determine the surgical effect on PLF-caused hearing loss.

The highly objective evidence rate of CTP-positive PLF in 70% of SSNHL patients, despite the initially recorded 90% idiopathic etiology of SSNHL in our study cohort, implies unclear events affecting inner ear structures. These findings imply a need for an early explorative tympanotomy with consecutive window sealing as a causative therapy. Further, long-term results are important to monitor fluctuation after the closure of the windows.

## 5. Conclusions

The combination of early tympanoscopy and inner ear-specific cochlin-tomoprotein as a detection tool for a suspected perilymphatic fistula shows evidence of PLF as a key causative in SSNHL. 

## Figures and Tables

**Table 1:** Individual data of patients of the study group.

Case No.	Age	Onset of Hearing Impairment before Surgery (in Days)	Vertigo (1 = yes 0 = no)	Intraoperative Subjective Evaluation of PLF	CTP Value	CTP Category	Etiology of SSNHL (Idiopathic/Traumatic)	Preoperative PTA Hearing Threshold (Max 3 Freq ^0 = 110 dB^)	Preoperative PTA Hearing Threshold (4 Freq ^0 = 130 dB^)	Postoperative PTA Improvement in dB (Max 3 Freq ^0 = 110 dB^)	Postoperative PTA Improvement in dB (4 Freq ^0 = 130 dB^)
1	52	2	0	negative	111.7	positive	idiopathic	66.6	65.0	25.6	27.5
2	48	3	1	positive	128.3	positive	idiopathic	110.0	130.0	0.0	0.0
3	61	7	0	negative	116.1	positive	idiopathic	103.0	105.0	−7.0	−25.0
4	44	4	0	negative	255.3	positive	idiopathic	110.0	130.0	0.0	30.0
5	89	4	1	negative	203.5	positive	idiopathic	108.3	107.5	3.3	1.3
6	18	6	1	positive	87.9	positive	traumatic	101.6	101.3	68.6	76.3
7	71	5	1	negative	32.5	intermediate	idiopathic	81.7	72.5	6.7	5.0
8	19	14	0	positive	58.1	intermediate	idiopathic	110.0	130.0	20.0	48.8
9	88	6	1	negative	80.5	positive	idiopathic	110.0	97.5	10.0	11.3
10	54	13	0	negative	58	intermediate	idiopathic	90.0	83.8	−20.0	−46.3
11	63	4	1	positive	134.8	positive	idiopathic	58.0	57.5	−7.0	−6.3
12	59	4	1	positive	41.9	intermediate	idiopathic	110.0	130.0	20.0	43.8
13	47	2	1	negative	100.5	positive	idiopathic	110.0	130.0	0.0	0.0
14	61	10	0	negative	186.1	positive	idiopathic	110.0	130.0	68.4	87.5
15	86	14	0	negative	244.6	positive	idiopathic	110.0	130.0	0.0	0.0
16	86	9	0	positive	53.4	intermediate	idiopathic	110.0	130.0	27.0	51.3
17	83	2	1	negative	24	negative	idiopathic	110.0	130.0	0.0	0.0
18	70	5	0	positive	61	positive	traumatic	110.0	130.0	28.4	55.0
19	21	1	1	negative	70.9	positive	idiopathic	110.0	130.0	20.0	53.8
20	38	6	1	positive	31.1	intermediate	idiopathic	110.0	130.0	0.0	35.0
21	55	3	1	positive	67.6	positive	traumatic	110.0	130.0	63.4	87.5
22	56	3	1	negative	133.6	positive	idiopathic	96.7	87.5	31.7	22.5
23	71	11	0	negative	32.5	intermediate	idiopathic	81.7	73.8	6.7	3.8
24	62	16	0	negative	165	positive	idiopathic	83.3	75.0	28.3	25.0
25	26	7	1	negative	119.2	positive	idiopathic	110.0	96.3	100.0	87.5
26	67	1	0	negative	174.7	positive	idiopathic	73.3	71.3	37.3	38.8
27	34	5	1	positive	184.6	positive	idiopathic	65.0	61.3	−10.0	−8.8
28	61	5	0	positive	129	positive	idiopathic	98.3	101.3	21.7	30.0
29	57	1	1	positive	144	positive	idiopathic	110.0	130.0	17.0	50.0
30	33	1	0	positive	57.8	intermediate	idiopathic	63.3	63.0	48.3	50.5

## Data Availability

Data are available from the corresponding author upon request due to containing information that could compromise the privacy of research participants.
